# Community-Based Dental Education (CBDE): A Survey of Current Program Implementation at Australian Dental Schools

**DOI:** 10.1155/2024/2890518

**Published:** 2024-07-04

**Authors:** Millicent Taylor, Sandra Carr, Omar Kujan

**Affiliations:** ^1^ Division of Health Professions Education School of Allied Health The University of Western Australia, Perth, WA, Australia; ^2^ UWA Dental School The University of Western Australia, Nedlands, WA, Australia

## Abstract

**Purpose:**

Community-based dental education (CBDE) diverges from traditional dental school training methods by integrating dental students into primary care community settings. This immersive approach enables students to refine their clinical and hands-on skills while serving the oral health needs of underserved populations. This study aimed to identify ways in which Australian dental schools are currently implementing CBDE and compared to current evidence.

**Materials and Methods:**

This study utilized a 24-item, self-completion survey, adapted from existing questionnaires, which was sent to the CBDE coordinators in the nine eligible dental programs in Australia between mid-January 2023 and mid-April 2023. The survey consisted of multiple-choice, binary, and open-ended questions, including information on the level of student involvement, types of external clinics used, length of rotations, student supervision and assessment, pre-rotation preparation, and post-rotation evaluation, as well as challenges faced in implementing programs.

**Results:**

Six of the nine invited coordinators responded, resulting in a 66.7% response rate. All participants confirmed that their schools had a community-based teaching program. All six respondents reported that participation in external clinics is required for graduation. Implementation of CBDE appears to be influenced by (1) level of student involvement, (2) the types of clinics utilised, (3) allocation and length of rotation, (4) student supervision and assessment, (5) pre-rotation preparation, and (6) post-rotation evaluation. Six (*n* = 6) institutions reported requiring a post-rotation reflection from students and all respondents reported seeking feedback from clinical supervisors at external sites. Emerging themes from open-ended questions highlight challenges in coordinating external rosters, securing funding, supervising students at external sites, and ensuring diverse types of student exposure during external rotations.

**Conclusion:**

This study provided insights into the implementation of CBDE in Australian dental schools. Results outlined in this research offer valuable insights for dental schools aiming to enhance their programs and improve student learning outcomes.

## 1. Introduction

Community-based dental education (CBDE) is an approach to teaching and learning in dentistry that takes place at a site or location outside the traditional dental education institution [1]. The main reason for this model of teaching is to provide students with real-world experience in primary care centers, contrasting the more complex needs typically encountered in patients at dental schools' secondary care facilities [1]. In the Australian context, primary dental care refers to the provision of dental services administered by general dental practitioners within community-based environments.

CBDE has gained recognition as an essential pedagogical methodology in dental training and plays a crucial role in preparing students for a career in dentistry [1, 2, 3, 4, 5, 6]. This educational approach aims to bridge the gap between theoretical knowledge and practical skills, nurturing well-rounded dental professionals who possess clinical competence, empathy, and a community-focused mindset [1, 2, 3, 4, 5, 6].

CBDE programs are typically collaborations between dental schools, public health services, community clinics, private dental practices, or public hospitals, creating a diverse and comprehensive learning environment [7, 8, 9]. By immersing students in these settings, they actively participate in patient care, diagnosis, treatment planning, and clinical procedures while supervised by experienced dental practitioners. This hands-on learning approach promotes critical thinking, problem-solving abilities, and clinical self-assurance [10, 11, 12].

CBDE programs vary based on the level of student involvement, length of rotations, the types of services provided, and the community partnership structures [7, 8, 9, 13]. The specific effects depend on the nature and structure of the programs implemented, for example, models that involve students providing dental care in community settings under supervision offer valuable hands-on clinical experience compared to observation-only rotations [6, 7, 8, 9, 13].

Furthermore, some CBDE programs incorporate opportunities for students to reflect on their experiences in community settings. This reflection allows students to analyze their clinical encounters, assess their strengths and weaknesses, and identify areas for improvement, leading to a deeper level of self-directed learning [14].

It is understood that different dental schools in Australia have adopted a range of community-based teaching strategies. Due to the evolving nature of CBDE programs, it is important to provide a current picture and comparison of how Australian dental schools employ CBDE in their curricula.

Therefore, this study aimed to:

1. Provide an overview of the current implementation of CBDE programs in Australian dental schools. This survey study sought to determine the types of external clinics utilized, the level of student involvement at clinics, length of rotations, pre- and post-rotation preparation and evaluation, and level of student supervision and assessment.
2. Compare results from this study to current international practices.

## 2. Materials and Methods

### 2.1. Study Design

The study is designed as a survey-based, cross-sectional descriptive study. Both open and closed questions were utilized to serve distinct purposes: while closed questions provided clarification and specificity, open-ended inquiries were used to explore deeper insights [15, 16].

### 2.2. Data Instrument

This study utilized a 24-item, self-completion survey, adapted from questionnaires developed by Mays [8] and Smith and Mays [5]. The research team reviewed and modified survey questions to suit local conditions (Appendix S1). The advantages of utilizing a pre-existing survey instrument are widely acknowledged, encompassing time efficiency, the use of a validated and reliable tool, and the ability to make comparisons with other studies that employed the same instrument [16].

The questionnaire sought information on the program's current structure. The survey questions were designed with a combination of closed and open styles. Closed style questions sought information on types of external clinics utilized, length of rotations, student supervision and assessment, clinical supervisor calibration, pre-rotation preparation, post-rotation feedback, and reflection. Respondents were invited to offer textual responses to open questions, particularly focusing on the challenges encountered by their institutions in implementing CBDE.

### 2.3. Participants

For this study, purposive sampling was employed, targeting program coordinators of CBDE programs at the nine Dental Schools in Australia. Purposive sampling is specifically utilized to identify and select participants with the most information possible and entails locating and choosing individuals or groups of individuals who have particular expertise in, or experience with an interest or phenomenon [17]. Purposive sampling in this study centers around the premise that CBDE program coordinators hold key responsibility for the CBDE program's implementation, making them valuable sources of focused information regarding the current practices at their respective institutions. In Australia, CBDE program coordinators are faculty members who typically manage both teaching and administrative responsibilities concurrently.

### 2.4. Recruitment

Introductory letters were sent to the heads of all nine Australian dental schools, giving a detailed overview of the research and requesting participation. Participating schools were asked to identify the members of faculty responsible for the CBDE program. Participants subsequently communicated directly with the principal investigator.

### 2.5. Data Collection

Secure online platform Research Electronic Data Capture (REDCap) was utilized to conduct the survey between mid-January 2023 and mid-April 2023.

### 2.6. Analysis Procedure

Each participant was de-identified and assigned an alphanumeric code. Coded transcripts on REDCap were exported directly to an Excel spreadsheet for statistical analysis, accessible to the principal investigator under a protective password.

Response options were dichotomous, multiple choice, or a textual response (Table 1).

Quantitative data were managed with descriptive statistics using frequencies and percentages to describe survey items (Table 2).

**Figure 1:**
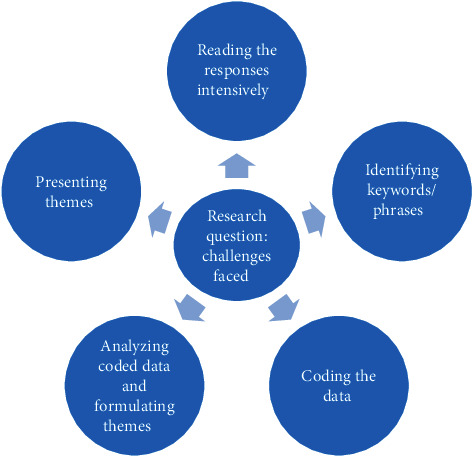
The five phases of qualitative content analysis (adapted from Kuckartz [18]).

Free text responses were analyzed, coded, and themes identified. The general workflow for the qualitative content analysis of the open-ended survey questions is illustrated in Figure 1, adapted from Kuckartz [18].

Utilizing the five phases of qualitative content analysis, the research question holds a central role [18]. It serves as the guiding perspective for the analysis, which involves thorough reading and analysis of the texts [18]. As customary in qualitative methodologies, the different phases of analysis were conducted in a cyclical manner, with the process of forming categories, coding the data, and formulating themes completed over an iterative process of multiple cycles [18].

## 3. Results

Of the nine eligible participants invited to participate in this study, six consented resulting in a 66.7% response rate. All (*n* = 6) confirmed that their schools had a community-based teaching program which provides opportunities for students to participate in external clinical rotations.

### 3.1. Types of External Clinics

**Figure 2:**
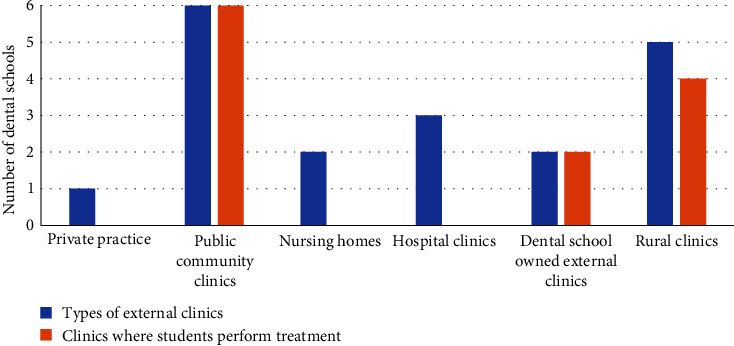
Types of external clinics.

When asked to categorize the type of external clinics affiliated with their school, the respondents stated that the sites were private practice (*n* = 1), public dental community clinics (*n* = 6), nursing homes (*n* = 2), hospital clinics (*n* = 2), dental school owned clinics (*n* = 2), public regional clinics (*n* = 6), and volunteer run dental clinics (*n* = 1) (Figure 2).

All participants indicated that they employ a combination of two models for community-based externships:

- The “dispersed practice” model, where students are allocated to selected community-based clinics, either in metropolitan (*n* = 6) or rural areas (*n* = 5).
- The “purpose built” model, which are bespoke university-owned, community-based primary care clinics staffed by the faculty (*n* = 2).

Three dental schools include external community rotations throughout their educational programs, not just limited to the final year. In their final year, students are typically assigned to public community clinics to gain hands-on experience in clinical treatments. Two schools send final year students to university-owned external clinics, while four schools offer opportunities to selected students at regional clinics. Observation-only rotations involve students attending hospital clinics (50%), private practices (17%), and nursing homes (33%), without administering treatment themselves (Figure 2).

### 3.2. Allocation and Length of Rotation

All dental schools implement a community-based teaching program typically in “blocks” during the final year of study, involving a specific number of uninterrupted weeks at outreach clinics. Two schools also incorporate a combination of block and “linear” teaching, which means continuous distribution throughout the year in earlier years of study. There is minimal variation in the time allocated to block rotations, with all participants (*n* = 6) rotating students to community clinics for 10 weeks or more. The number of weeks allocated to block rotations per institution ranges from 13 to 34 weeks, with one school rotating students for the full academic year at external sites.

### 3.3. Student Supervision and Assessment

Participation in external rotations is a graduation requirement for most (*n* = 5) schools. Three schools reported that students are supervised and assessed solely by clinicians from the external organization, who are not necessarily trained as assessors nor calibrated for reliability. Conversely, in three institutions, faculty members and external clinicians collaborate in supervising and evaluating students, with external clinicians undergoing training and calibration at the dental school to ensure consistent student assessments. All institutions reported that students are required to complete a self-reflection of their experiences at external sites as a component of their assessment.

### 3.4. Pre-rotation Preparation

All schools reported that students are provided with a structured orientation and site manual at external sites where students undertake dental treatment. Five respondents noted that their institution had an affiliate agreement or memorandum of understanding (MoU) with the external organization, while one respondent was unsure. However, none of the respondents provided details about the content of these agreements.

### 3.5. Post-rotation Evaluation

Five institutions reported performing a post-rotation survey to seek students' perspectives, and all respondents reported seeking formal feedback from clinical supervisors at external sites. Four respondents reported conducting site visits to external clinics, 1−2 per year, while three institutions indicated that site visits are not conducted.

### 3.6. Challenges Faced in Implementing CBDE

Two free-text questions were presented to participants. Participants were requested to share their insights regarding:

- challenges faced by their institutions in administering CBDE/outreach teaching programs, and
- to add additional comments.

Five of the six participants provided responses to the open-ended questions.

Results of applying the standard inductive thematic analysis to free text survey questions found that comments provided by participants were related to four emerging themes (Table 3).

## 4. Discussion

Results from this study indicate that most Australian Dental Schools include CBDE in their curriculum. Program implementation appears influenced by the following: (1) level of student involvement, (2) the type of external clinic, (3) allocation and length of rotation, (4) student supervision and assessment, (5) pre-rotation preparation, and (6) post-rotation evaluation.

### 4.1. Level of Student Involvement

It is evident that students are rotated to external sites throughout their studies. However, it is predominantly in the final year of study that students attend external rotations where they are involved in performing dental treatment on patients. All sites where students perform dental treatments are within primary care settings.

Current evidence suggests that CBDE models that involve students providing dental care in community settings, under supervision, offer valuable hands-on clinical experience [10, 11, 12].

This exposure has been shown to enhance students' technical skills, critical thinking abilities, and problem-solving aptitude, ultimately improving their clinical competence and readiness for independent practice [10, 11, 12]. Additionally, by engaging with underserved populations and witnessing oral health disparities, students develop a heightened sense of social responsibility and an understanding of primary care dentistry's role in public health [3]. These attributes align with the requirements set by the Australian Dental Council (ADC) [19] for newly graduated dentists.

### 4.2. Types of External Clinics

In this study, community dental clinics are the most frequently utilized for final year dental student skills refinement, followed by rural-based clinics.

While no studies have compared educational outcomes between dispersed and purpose-built community clinics, Lynch et al. [9] speculate that dental schools' choice to utilize purpose-built units may stem from perceived control over staffing, funding, educational content, and program coordination. Another reason for utilizing bespoke units is to gain direct access to appropriate patients in primary care settings, eliminating the need to rely on referrals for care at teaching hospitals [1, 7, 13].

The dispersed model, conversely, also has its advantages. First, it is cost-effective for universities by utilizing existing community dental clinics, reducing the need for additional resources [9]. Second, community dentistry clinics typically serve a higher volume of patients compared to academic clinics, providing students with more opportunities to practise clinical skills [1, 8, 13]. Lastly, partnerships with public dental clinics enhance the university's reputation and promote goodwill within the faculty and the community [20, 21].

Four universities in this study indicated offering rotations for students in rural locations. This is encouraging considering the critical skills shortages encountered in rural areas in Australia [22]. Placing students in rural areas for clinical rotations offers various benefits, including exposure to less common patient cases, broadening student skill set, increased clinical confidence, enhanced community engagement, cultural competence development, opportunities for interdisciplinary collaboration, and potential rural employment after graduation [23, 24, 25, 26, 27].

However, rural placements also present unique challenges such as limited funding, administrative burdens, limited resources including dental equipment and facilities, shortage of experienced dentists for supervision, and student isolation from usual support networks [24, 25, 28]. Qualitative data from this study confirm that securing funding and organizing rural placements are significant challenges for program coordinators (Table 3).

### 4.3. Allocation and Length of Rotation

Depending on the curriculum of the dental school, CBDE rotation lengths and allocations are varied in this study. The general trend noted in this study is that urban dental schools with a traditional curriculum offer shorter community-based rotations compared to longer rotations offered by dental schools with a rural-based program. The qualitative findings of this study suggest that program coordinators encounter difficulties in arranging suitable placements, particularly for short and rural rotations, which present notable administrative challenges.

Block allocations and linear allocations are two different approaches in assigning students to external rotations [29, 30]. In block allocations, students are assigned to outreach rotations by timeframes, such as weeks or months and all students within a particular block undergo the same clinical experiences simultaneously before rotating to a different experience in the next block [29, 30]. In a linear allocation, students are assigned to an external rotation on an ongoing basis, usually offered in the earlier years of study [29, 30]. The merits or limitations of either allocation are primarily determined by the logistical challenges of coordinating placements [29, 30].

Block rotations can vary from a few weeks to several months, influenced by curriculum requirements, rotation aims, and accessibility of community sites [1, 13, 29]. The duration of rotations has several impacts on the CBDE program and outcomes. Shorter rotations, typically lasting three to 4 weeks, allow students to briefly familiarize themselves with community settings and gain understanding of oral health needs in underserved areas [31]. However, such short-term rotations present with several disadvantages on rotations where students perform dental treatment on patients [31, 32]. Studies indicate that short CBDE rotations are not long enough to allow significant learning experiences for students and are insufficient in fostering opportunities for reflection [8, 13, 32]. Mascarhenas et al. [32] report that it takes students 2 weeks to adjust to a new clinical environment. In addition, short rotations can negatively impact the continuity of patient care and strain relationships with community partners [8, 32]. Due to the time required for more comprehensive treatment, short rotations limit the type of patient encounters community partners can accommodate and consequently limit student skill development [8, 31, 32]. On short-stay rural rotations, where longer placements may not be logistically possible, dental schools and clinical partners are required to work together to ensure appropriate handovers and seamless transitions of care [31].

Conversely, longer rotations, often in the final year of study and lasting eight uninterrupted weeks or longer, enable students to immerse themselves in clinical practice and provide comprehensive care to patients over an extended period [8, 13, 32]. This may also be beneficial for the community partner sites as longer student rotations have been proven to increase student clinical productivity [8, 13, 32].

While longer rotations offer profound learning opportunities, enhanced competencies, and stronger community connections, they require careful preparation to address logistical challenges and maintain a comprehensive dental curriculum [8, 13, 29, 30, 32].

### 4.4. Student Supervision and Assessment

Most dental schools in this study allow competency assessments at external locations which contribute to graduation. However, not all offer training and calibration for the assessors. This study's findings indicate that variations exist among dental schools in the training, calibration, and support provided to the community-based clinical supervisors. Moreover, results from open-ended questions in this study suggest that differences in educational approaches between faculty and partner sites, as well as variations in student supervision at external sites, present as substantial barriers in implementing CBDE programs.

Community clinics primarily prioritize patient care, leading to significant differences in the educational approach compared to academic faculty settings [4, 7, 33, 34]. Typically, clinical supervisors at community partner sites are general dentists who may not have intentionally pursued a teaching career resulting inevitable challenges [33, 34]. In a studies by Smith et al. [21] and Bartle and McGowan [33], community student supervisors expressed challenges in balancing expected clinical obligations prescribed by their employer with the training requirements of students. In addition, clinical supervisors experienced difficulty in communicating with the university regarding student competence and professionalism, as well as keeping students interested and motivated [21, 33, 34].

Furthermore, community student supervisors are frequently tasked with conducting competency assessments of students [20, 21, 33]. These studies underscore the importance of ensuring that assessors at community sites receive adequate training and calibration to effectively evaluate student competencies [20, 21, 33].

It is therefore recommended that community clinical supervisors undergo training at the dental school in order to provide appropriate supervision, reliable assessments, and ensure cohesion between the community site's service goals and the university's educational goals [20, 21, 33, 34].

All participants in this study indicated that students must conduct self-reflections on their experiences at external sites as part of their assessment. Student reflection is integral to service learning, highlighting that CBDE should not only enhance clinical skills but also promote personal and professional development [2, 13, 14]. Reflection enables students to connect community service with academic goals, explore social issues, and contemplate personal values [14]. According to Strauss et al. [2] reflections following CBDE rotations should encompass critical incident reports, reflective essays, and portfolios documenting treatments and experiences.

### 4.5. Pre-rotation Preparation

A memorandum of understanding (MOU) or affiliate agreement between the university and community partners is of significant importance in the context of CBDE and its implementation is emphasized in current literature [7]. This agreement should be in place prior to commencing any CBDE program and cover the responsibilities and obligations of both organizations [7].

Pre-rotation preparation involves several key components to ensure students are adequately prepared for their external placements. First, dental schools should ensure that students possess the core competencies before their rotation, including foundational clinical skills and professionalism [7, 13]. In addition, Yoder [13] emphasizes that students be familiarized with the mission and values of the external site along with the expectation that they comply with the policies and protocols of the host service. Mandatory orientation sessions should cover placement objectives, site expectations, characteristics of the patient population, protocols for clinical procedures, clear guidelines for supervised practice, documentation of patient interactions, and clinic workflow, as well as outlining common issues encountered in community settings [7, 13, 29].

Second, it is important to provide students with a policy and procedure manual tailored specifically for student placements, which should be developed by partner sites [7, 13].

Lastly, dental schools should provide partner sites with appropriate and timely information, including student profiles outlining skills and competencies, at least 4 weeks before the rotation begins [7]. This allows site coordinators to plan for students, schedule patients, and organize staffing effectively [7].

### 4.6. Post-rotation Evaluation

Community partner organizations play a vital role in identifying the issues targeted by the program [7, 13, 33]. When selecting service-learning activities, it is essential to strike a balance between the educational needs of students and the needs of the community being served [7, 13, 21, 33]. Gathering formal feedback from both students and clinical supervisors to gain insights into challenges and needs at outreach settings is crucial for improving CBDE programs [7, 13].

Furthermore, post-rotation site visits by faculty members offer an opportunity to clarify goals, strengthen roles, and review teaching and assessment principles [20, 33].

## 5. Conclusion

This descriptive study provided insights into the implementation of CBDE in Australian dental schools. This seems to be influenced by (1) level of student involvement, (2) the types external clinics, (3) allocation and length of rotation, (4) student supervision and assessment, (5) level of pre-rotation preparation, and (6) level of post-rotation program evaluation.

There are two encouraging trends emerging from this study: first, all participating universities seek formal feedback from clinical supervisors at external sites to enhance the program, and second, the duration of rotations is planned to be mutually beneficial for students and external clinics.

Nevertheless, findings and existing evidence suggest that increased collaboration is necessary between dental schools and affiliated sites to train, align, and support clinical supervisors at community clinics.

Although it is acknowledged that there is no singular approach to CBDE, the findings identified in this study for effective CBDE implementation aim to serve as a guide for dental schools looking to strengthen their programs and improve student learning outcomes.

## Figures and Tables

**Table 1:** Survey questions and response type for data collected in study.

Question scope	Question	Response type
Availability of CBDE at institution	(1) Does your institution offer opportunities for students to participate in external clinical rotations?	Dichotomous

Types of external clinics	(2) Please identify the types of clinics where students attend external rotations (tick all that apply)	Multiple choice
	(3) Please specify if option “Other” was selected	Textual response
	(4) Please identify the sites where students perform clinical procedures themselves, under supervision, during their external rotation (tick all that apply)	Multiple choice
	(5) Please specify if option “Other” was selected	Textual response

Allocation and length of rotation	(6) In what year of the students' dental training do they rotate out to external sites where they perform clinical procedures	Written response
	(7) Are students allocated to external clinics in “blocks” or as a linear distribution throughout the year?	Dichotomous
	(8) For “block” rotations, please specify number of weeks per academic year	Textual response
	(9) For linear distribution, please specify the approximate number of days per academic year	Textual response

Student supervision and assessment	(10) Do students receive clinical credit toward graduation for procedures performed at external sites?	Dichotomous
	(11) Please specify who is responsible for student supervision at external sites where students perform clinical procedures?	Multiple choice
	(12) Additional information to previous question	Textual response
	(13) Please specify who is responsible for on-site competency assessments of students at external sites	Multiple choice
	(14) Additional information to previous question	Textual response
	(15) Are student supervisors at external sites calibrated?	Dichotomous

Pre-rotation preparation	(16) Are students provided with a structured orientation and site manual at these sites?	Dichotomous
	(17) Does your institution have a memorandum understanding (MoU) or affiliate agreement with external site organisations?	Dichotomous

Post-rotation evaluation	(18) Does your institution perform a post-rotation survey of students' perspectives of their experience at external sites?	Dichotomous
	(19) Do students complete a self-reflection of their experience at external sites?	Dichotomous
	(20) Does the faculty seek input/feedback from student supervisors at external sites?	Dichotomous
	(21) Does the faculty conduct site visits to external sites where students perform clinical procedures?	Dichotomous
	(22) If “yes” to previous question, how many times a year?	Textual response

Challenges faced in implementing CBDE	(23) What are the challenges faced by your institution in administering outreach/community-based dental education at external clinics?	Free text responses
	(24) Are there any other comments you would like to add?	

**Table 2:** Survey results.

Questions	Answer	*n*	Percentage
(1) Does your institution offer opportunities for students to participate in external clinical rotations?	Yes	*n* = 6	100
	No	*n = 0*	

(2) Please identify the types of clinics where students attend external rotations (tick all that apply)	Private practice	*n = 1*	17
	Public dental community clinics	*n = 6*	100
	Nursing homes	*n = 2*	33
	Hospital clinics	*n = 3*	50
	Dental school owned external clinics	*n = 2*	33
	Public regional clinics	*n = 5*	83
	Other	*n* = 0	

(3) Please specify if option “Other” was selected	—	None	None

(4) Please identify the sites where students perform clinical procedures themselves, under supervision, during their external rotation (tick all that apply)	Private practice	*n = 1*	17
	Public dental community clinics	*n = 6*	100
	Nursing homes	*n = 1*	17
	Hospital clinics	*n = 3*	50
	Dental shool owned external clinics	*n = 2*	33
	Public regional clinics	*n* = 4	66.7
	Other	*n* = 0	

(5) Please specify if option “Other” was selected	—	None	None

(6) In what year of the students' dental training do they rotate out to external sites where they perform clinical procedures	4 (final year)		
	3, 4, 5		
	3		
	5 (final year)		
	5 (final year)		
	3, 4, 5		

(7) Are students allocated to external clinics in “blocks” or as a linear distribution throughout the year?	Blocks	*n* = 4	66.7
	Linear	*n = 0*	
	Both	*n = 2*	33

(8) For “block” rotations, please specify number of weeks per academic year	15 weeks		
	17 weeks		
	34 weeks		
	13 weeks		
	12–14 weeks (final year)		
	Full academic year, 4 days/week		

(9) For linear distribution, please specify the approximate number of days per academic year	Final year (70 days)	—	—

(10) Do students receive clinical credit toward graduation for procedures performed at external sites?	Yes	*n* = 5	83
	No	*n = 1*	17

(11) Please specify who is responsible for student supervision at external sites where students perform clinical procedures?	Students are supervised by Faculty staff	*n = 2*	33
	Students are supervised by clinicians employed by external site organization	*n* = 6	100
	Other	*n = 1*	17

(12) Additional information to previous question	—	—	—

(13) Please specify who is responsible for on-site competency assessments of students at external sites.	Students assessed by faculty member	*n = 2*	33
	Students assessed by clinicians employed by external site organization	*n* = 6	100
	Other	*n* = 1	

(14) Additional information to previous question	None	—	—

(15) Are student supervisors at external sites calibrated?	Yes	*n = 3*	50
	No	*n* = 3	50
(16) Are students provided with a structured orientation and site manual at these sites?	Yes	*n = 6*	100
	No	*n* = 0	

(17) Does your institution have a memorandum understanding (MoU) or affiliate agreement with external site organizations?	Yes	*n = 5*	83
	No	*n* = 0	17
	Not sure	*n = 1*	

(18) Does your institution perform a post-rotation survey of students' perspectives of their experience at external sites?	Yes	*n = 4*	66.7
	No	*n* = 2	33

(19) Do students complete a self-reflection of their experience at external sites?	Yes	*n = 6*	100
	No	*n* = 0	

(20) Does the faculty seek input/feedback from student supervisors at external sites?	Yes	*n = 6*	100
	No	*n* = 0	

(21) Does the faculty conduct site visits to external sites where students perform clinical procedures?	Yes	*n = 4*	66.7
	No	*n* = 2	33

(22) If “yes” to previous question, how many times a year?	1		
	1–2		
	1–2		
	2		

(23) What are the challenges faced by your institution in administering outreach/community-based dental education at external clinics?	See Table 3	—	—

(24) Are there any other comments you would like to add?	See Table 3	—	—

**Table 3:** Thematic matrix of open-ended survey questions.

Emerging theme	Response	Commentary
Theme 1: Challenges regarding coordinating external rosters	“Allocating students to attend rural remote placements, where students have family/medical other reasons to remain local”; “As coordinator, the challenge is to ensure placements and students feel supported at all times”; “The relatively short period of placement. Would be more beneficial if students get a full year of placements”	This emerging theme suggests that coordinating schedules and ensuring appropriate placements, specifically on short and rural rotations, pose an administrative burden for coordinators

Theme 2: Challenges regarding funding	“Primarily the costs associated with outreach placements: costs of travel, accommodation”; “Funding is the biggest challenge, particularly at university operated rural clinics”; “There has to be discussion for clinical placements to be adequately funded”; “The limited number of seats available for students' placements”	This emerging theme suggests that coordinators encounter difficulties in securing funding, especially for rural placements

Theme 3: Challenges regarding student supervision at external sites	“Student experience can be impacted by the quality of clinical supervisors in metro clinics operated by external partners”; “Supervision at these sites is therefore non-standardised, students have differing experiences”; “The gap between educational styles in university clinics and outplacement clinics and the absence of support to bridge the gap”; “Staffing where the school is responsible for providing staff”; “Finding appropriate clinics with external supervision”; “Getting educators on board university policies such as using Pebblepad for student marking as they are not direct employees of the university”	This emerging theme suggests that differing educational approaches between faculty and partner sites pose a challenge for program coordinators

Theme 4: Challenges regarding types of student exposure on external rotations	“Students exposed to mainly relief of pain type of dental treatments”; “Some variation in the range of clinical services provided—mainly public health system”; “It would be good is students can collaborate with GPs and other health professionals during their rural posting”	This emerging theme suggests that coordinators consider the types of clinical exposure students encounter in a primary care public health setting a challenge

## Data Availability

The data that support the findings of this study are openly available in the University of Western Australia research repository at https://doi.org/10.26182/azts-w959.

## Additional Points

*Limitations*. A limitation of this study was the challenge of identifying the most knowledgeable participant at each dental school regarding the CBDE program. This could have influenced the interpretation and accuracy of responses if the survey did not reach the most informed person. Additionally, while six out of a potential nine participants responded, the missing participants may have introduced sampling bias. These limitations are consistent with findings from similar studies [8, 9]. It should be noted that while the sample size is adequate for Australian dental schools, its applicability on a global scale may be limited. Current evidence indicates that community partners hosting CBDE programs stand to gain several advantages. These include the opportunity to mentor students for prospective employment, fostering community awareness among future dental practitioners, and staying abreast of advancements in clinical dentistry through university collaboration [20, 21, 23, 34]. Likewise, scholarly literature extensively documents the opportunities for both professional and personal development afforded to students engaged in CBDE programs [2, 3, 4, 5, 10, 12, 14]. However, the primary objective of this study is to offer a baseline overview of the present state of CBDE implementation in Australia from the Faculty perspective. Therefore, the authors acknowledge that there are limitations in the scope of this study, such as the perspectives of students and community partners in Australia, highlighting the need for further research to achieve a more comprehensive understanding.

## Supplementary Materials

Supplementary Materials Appendix S1: contains the survey instrument utilized in this research study.
