# Supplementary material for: Community-Based Dental Education (CBDE): A Survey of Current Program Implementation at Australian Dental Schools
Source: Int J Dent. 2024 Jul 4;2024:2890518. doi: 10.1155/2024/2890518 (PMC11239228; doi:10.1155/2024/2890518)
Supplement: Supplementary Materials — Appendix S1: contains the survey instrument utilized in this research study. [file 2890518.f1.docx]

**Appendix 1: Survey Instrument** Adapted from Mays, 2016^8^ and Smith and Mays, 2019^5^

**Survey of Community Based Dental Education (CDBE) Experiences**

Does your institution offer opportunities for students Yes

to participate in external clinical rotations? No


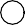

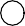


Please identify the types of clinics where students attend external rotations (tick all that apply).

Private Practice

Public dental community clinics Nursing homes

Hospital clinics

Dental School owned external clinics (purpose built)

Public regional clinics Other

For 'Other', please specify

Please identify the sites where students perform clinical procedures themselves, under supervision, during their external rotation (tick all that apply).

Private practice

Public dental community clinics Nursing homes

Hospital clinics

Dental School owned external clinics (purpose built)

Public regional clinics Other

For 'Other', please specify

In what year of the students' dental training do they rotate out to external sites where they perform clinical procedures?

Are students allocated to external clinics in "blocks" or as a linear distribution throughout the year?

Blocks

Linear distribution

For 'Block' rotations, please specify number of weeks per academic year

For linear distribution, please specify the approximate number of days per academic year

Do students receive clinical credit towards graduation Yes

for procedures performed at external sites? No


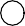

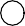


Does your institution perform a post rotation survey Yes

of students' perspectives of their experience at No external sites?


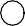

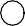


Do students complete a self-reflection of their Yes

experience at external sites? No


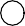

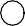


Are students provided with a structured orientation and site manual at these sites?

Yes No

Not sure

Please specify who is responsible for student supervision at external sites where students perform clinical procedures?

Students are supervised by Faculty staff

Students are supervised by clinicians employed by external site organisation

Other

Additional information to previous question (can be left blank)

Please specify who is responsible for on-site competency assessments of students at external sites.

Students assessed by Faculty member Students assessed by clinicians employed by external site organisation

Other

Additional information to previous question (can be left blank)

Are student supervisors at external sites calibrated?

Yes No

Does the Faculty seek input / feedback from student supervisors at external sites?

Yes No

Does the Faculty conduct site visits to external sites where students perform clinical procedures?

Yes No

If 'yes' to previous question, how many times a year?

Does your institution have a Memorandum of Understanding (MoU) or Affiliate agreement with external site organisations?

Yes

No

Not sure

What are the challenges faced by your institution in administering outreach / community-based dental education at external clinics?

Are there any other comments you would like to add?
